# Cell-surface nucleolin is sequestered into EPEC microcolonies and may play a role during infection

**DOI:** 10.1099/mic.0.047506-0

**Published:** 2011-06

**Authors:** Paul Dean, Brendan Kenny

**Affiliations:** Institute for Cell and Molecular Biosciences, Medical School, University of Newcastle, Framlington Place, Newcastle upon Tyne NE2 4HH, UK

## Abstract

Nucleolin is a prominent nucleolar protein that is mobilized into the cytoplasm during infection by enteropathogenic *Escherichia coli* (EPEC). Nucleolin also exists at low levels at the cell surface of eukaryotic cells and here we show that upon infection of an intestinal cell model, EPEC recruits and subsequently sequesters cell-surface EGFP-nucleolin into extracellularly located bacterial microcolonies. The recruitment of nucleolin was evident around bacteria within the centre of the microcolonies that were not directly associated with actin-based pedestals. Incubation of host intestinal cells with different ligands that specifically bind nucleolin impaired the ability of EPEC to disrupt epithelial barrier function but did not inhibit bacterial attachment or other effector-driven processes such as pedestal formation or microvilli effacement. Taken together, this work suggests that EPEC exploits two spatially distinct pools of nucleolin during the infection process.

## Introduction

Enteropathogenic *Escherichia coli* (EPEC) is a non-invasive pathogen that binds to human small intestinal enterocytes and delivers multiple effector proteins into host cells via its type III secretion system (Dean & Kenny, 2009). These effectors subvert many aspects of host cell physiology, ultimately leading to diarrhoeal disease (Dean & Kenny, 2009). EPEC poses a significant threat in developing countries and is a leading cause of infantile diarrhoea (Chen & Frankel, 2005). Although the functions of EPEC effectors are becoming determined, the interaction of EPEC with the host plasma membrane is less clear. EPEC does not typically enter host intestinal cells but forms three dimensional microcolonies on the host cell surface mediated by the bundle-forming pilus (Nougayrède *et al.*, 2003). As physical contact between the bacterium and the host cell membrane is required for the delivery of the effectors, it follows that many individual bacteria within the attached microcolony will not be engaged in the delivery of effectors.

EPEC belongs to a family of pathogens that include enterohaemorrhagic *E. coli* (EHEC) and *Citrobacter rodentium*. These pathogens all share the LEE (locus of enterocyte effacement) genomic pathogenicity island, which encodes several important virulence factors including the type III secretion system, six effectors and the outer-membrane protein intimin. The effector Tir (translocated intimin receptor) is the best-studied LEE effector; it inserts into the host plasma membrane, where it acts as a receptor for intimin and facilitates bacterial attachment (Kenny *et al.*, 1997). Upon binding intimin, Tir induces extensive actin polymerization within the host cell, leading to the formation of an actin-rich pedestal (Kenny *et al.*, 1997).

Recently, another LEE effector, EspF, was shown to cause extensive mobilization of the nucleolar protein nucleolin from the nucleolus into the cytoplasm during late-stage infection (Dean *et al.*, 2010; Holmes *et al.*, 2010). Nucleolin is the most abundant protein in the nucleolus, where it plays important roles in ribosome biogenesis, although several studies have also demonstrated the presence of nucleolin at the cell surface by antibody detection (Christian *et al.*, 2003; Hovanessian *et al.*, 2000; Losfeld *et al.*, 2009; Nisole *et al.*, 1999; Storck *et al.*, 2007; Watanabe *et al.*, 2010). However, given that nucleolin-like proteins have also been detected at the cell surface (de Verdugo *et al.*, 1995; Kleinman *et al.*, 1991; Krantz *et al.*, 1995), concerns have been raised regarding the use of antibodies to detect cell-surface nucleolin (Ginisty *et al.*, 1999). Immunodetection was used to demonstrate that cell-surface nucleolin is recruited *in vivo* by EHEC to the vicinity of the bacterial attachment site (Sinclair *et al.*, 2006), where it is believed to act as a bacterial adhesin (Sinclair & O'Brien, 2002). Indeed, nucleolin has been shown to directly bind the EPEC and EHEC outer-membrane protein intimin (Sinclair & O’Brien, 2002) although the relevance of this during infection of host cells is less clear as intimin was not shown to colocalize with nucleolin during EPEC/EHEC infection of Hep-2 cells (Sinclair & O'Brien, 2004). Thus, the nature of nucleolin recruitment by EHEC and the importance of this during EPEC infection remain to be determined.

In the present study, we analysed the nature of nucleolin recruitment during EPEC infection of intestinal cells by expressing enhanced green fluorescent protein (EGFP)-nucleolin in the small intestinal Caco-2 model. We reveal that nucleolin is not only recruited to the EPEC infection site but is also sequestered transiently inside extracellularly located bacterial microcolonies. We present data to suggest that cell-surface nucleolin is involved in the EPEC infection process but does not significantly contribute to bacterial attachment or effector delivery. Taken together, the work shows that EPEC exploits two spatially distinct pools of nucleolin: the main nucleolar pool during late-stage infection and a cell-surface pool at earlier infection times.

## Methods

### 

#### Cell line, plasmid and bacterial strain.

Caco-2 cells were cultured and transiently transfected with pEGFP-nucleolin as described previously (Dean *et al.*, 2010). Infection experiments were performed with the prototypical EPEC strain E2348/69. This strain carries the EAF plasmid, which enables the bacteria to form compact microcolonies on infected host cells.

#### Confocal microscopy.

For microscopy, all transfected cells (5–7 days post-seeding) were infected with EPEC E2348/69 at an m.o.i. of 1 : 50 using standard infection procedures (Dean & Kenny, 2004) and processed for confocal microscopy as described previously (Dean *et al.*, 2010). Cells were stained for filamentous actin (TRITC-phalloidin) and DNA (DAPI) and viewed on a Leica SP2 confocal microscope. Pedestal formation (the polymerization of actin underneath the bacteria) is a diagnostic indicator of EPEC infection and was used, along with DAPI, to identify EPEC infection sites. Image deconvolution was performed with Huygens professional deconvolution software. Immunofluorescence was performed using the nucleolin antibody MS-3 (Santa Cruz Biotechnology) as described by Sinclair & O'Brien (2004) or ab22758 (Abcam) as described by Dean *et al.* (2010). Caco-2 cells were processed in a variety of ways to try to visualize cell-surface nucleolin by immunofluorescence, including fixation with 2–4 % paraformaldehdye or methanol, permeabilization using 0.1 % Tween 20, 0.2 % Triton X-100 or 0.1 % saponin, or not permeabilized at all. In all cases, and despite a strong nuclear signal of nucleolin, cell-surface nucleolin was not detected by immunofluorescence on these cell types (not shown).

#### Nucleolin ligand-binding assays.

Differentiated Caco-2 cells (15 days post-confluence) were exposed to different concentrations of midkine or pleiotrophin (R and D systems) for 3 h prior to infection with wild-type EPEC as described by Dean & Kenny (2004). Procedures relating to transepithelial resistance (TER) and scanning electron microscopy have been described previously (Dean & Kenny, 2004; Dean *et al.*, 2006). Confocal microscopy was used to determine the number of attached bacteria following three washes in PBS and DAPI staining. Pedestal formation was assessed by fluorescent actin staining using phalloidin as described by Dean & Kenny (2004). Neither midkine nor pleiotrophin had any observable effects on the appearance of host cells or on TER values (not shown).

#### Statistical analyses.

Means were compared by ANOVA or Student’s *t*-test using the statistical package SPSS, with *P*-values below 0.01 taken as significant.

## Results

### EPEC transiently recruits cell-surface nucleolin during infection of intestinal cells

Previous studies have shown that EHEC recruits cell-surface nucleolin to the vicinity of the bacterial infection site on cell lines (Sinclair & O'Brien, 2004) and during *in vivo* infection (Sinclair *et al.*, 2006). However, although nucleolin has been shown to bind directly to the EHEC outer-membrane protein intimin, it was not found to colocalize with the bacterial outer membrane during EPEC/EHEC infection of host cells (Sinclair & O'Brien, 2004) and thus the role of nucleolin and the nature of nucleolin recruitment during infection remain undetermined.

**Fig. 1.**
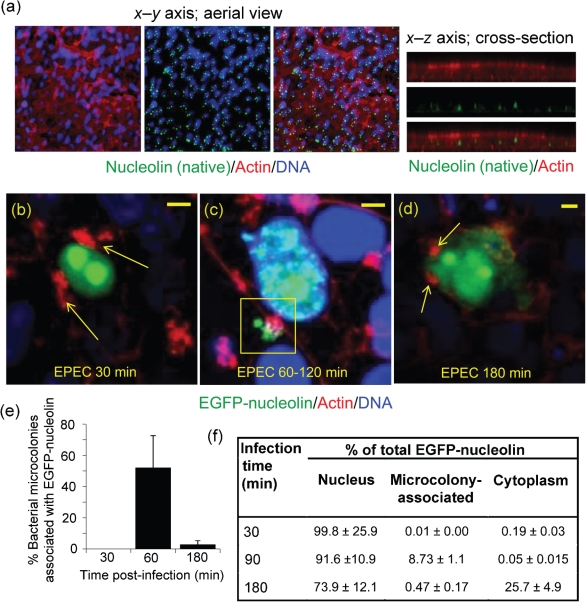
EPEC recruits EGFP-nucleolin at the cell surface. (a) Immunofluorescence of confluent Caco-2 cells stained for nucleolin and visualized along the *x*–*y* (aerial view) or *x*–*z* axis (cross-section). (b–d) Caco-2 cells (5–7 days post-confluence) expressing EGFP-nucleolin infected with EPEC for (b) 30 min, (c) 60–120 min and (d) 180 min. Arrows in (b) and (d) indicate bacterial-associated actin pedestals. The square in (c) indicates microcolony-associated EGFP-nucleolin. Yellow scale bars, 5 µm. Bacteria appear blue in all images. (e) Percentage of bacterial microcolonies assocated with EGFP-nucleolin signal at different infection times. Microcolonies were counted only on host cells expressing EGFP-nucleolin, and a region of interest around each microcolony was made and assessed for the EGFP levels above the background signal. Bars show mean±sem (*n* = 3). (f) Quantification of the subcellular EGFP-nucleolin signal. The nucleolin fluorescence signal was quantified using confocal software. Regions of interest were detected as follows: the host nucleus was determined using DAPI; the cytoplasm was the region between the host nucleus and the cell peripheral actin stain; microcolonies were located using the bacterial DAPI stain. The nucleolin signal in different fractions is expressed as a percentage of total cell fluorescence. Values are means±sem (*n* = 3).

Recently, we have shown that nucleolin within the host-cell nucleus is significantly disrupted following prolonged EPEC infection (Dean *et al.*, 2010) and this analysis was extended to assess nucleolin’s behaviour at the cell surface of intestinal cells. Using two different nucleolin antibodies (see Methods) that have been used previously to detect both nuclear (Dean *et al.*, 2010) and cell-surface nucleolin on Hep-2 cells (Sinclair & O'Brien, 2004), we were unable to detect cell-surface nucleolin on intestinal Caco-2 cells despite observing nucleolar nucleolin with these antibodies (Fig. 1a). Indeed, a range of fixation and permeabilization methods were employed (see Methods) in an attempt to detect cell-surface nucleolin by immunofluorescence (IF) but these were unsuccessful (data not shown), suggesting that the expression of native nucleolin on the surface of Caco-2 cells may be below detection levels by IF, despite being detected in cell fractions of Caco-2 cells by Western blot analysis (Dean *et al.*, 2010). This low level of nucleolin expression at the cell surface is in line with previous *in vivo* findings using mouse, calf and piglet intestinal sections (Sinclair *et al.*, 2006). Thus, because of the weak IF signal with intestinal Caco-2 cells, we employed an overexpression system using an EGFP-nucleolin fusion expressed in the Caco-2 cell line to follow the behaviour of cell surface nucleolin during EPEC infection.

Confocal analysis of Caco-2cells (5–7 days differentiated) that were transiently transfected with EGFP-nucleolin revealed that in agreement with the IF data (Fig. 1a), nucleolin was only detected in the nucleolus/nucleus of uninfected cells, with no observable accumulation at the cell surface – consistent with previous reports on other cell types (Chen & Huang, 2001; Dean *et al.*, 2010). This nucleolar/nuclear localization pattern of EGFP-nucleolin remained unaltered following a 30 min infection with EPEC (Fig. 1b) but by 60–120 min, it strongly colocalized with the EPEC microcolonies on the surface of transfected Caco-2 cells (Fig. 1c). Although recruitment of cell-surface EGFP-nucleolin was a prominent event, not all bacterial microcolonies were engaged in recruiting EGFP-nucleolin (~50 %; Fig. 1e). By 180 min post-infection, cell-surface EGFP-nucleolin associated with attached bacteria (detected by DAPI and actin pedestals) had diminished to near background levels (Fig. 1d), suggesting that nucleolin recruitment was a transient event. This loss of recruited EGFP-nucleolin coincided with the mobilization of nucleolin into the cytoplasm (Fig. 1d) and the loss of nucleolin from the membrane fraction of Caco-2 cells, as reported previously (Dean *et al.*, 2010). These microscopy findings were supported by quantification of microcolony-associated EGFP-nucleolin (Fig. 1e) and subcellular location of the EGFP-nucleolin signal during infection (Fig. 1f), which provided further evidence that nucleolin recruitment was a transient event. Cell surface nucleolin was found to be intimately associated with EPEC microcolonies (see below), but it was unclear whether the transience of cell-surface recruitment was related to changes in microcolony dynamics during the infection process.

As EPEC infection caused highly localized recruitment of EGFP-nucleolin at the cell surface, we tested whether native nucleolin could be visualized at the cell surface of infected cells by IF of infected cells. A small increase in native cell-surface nucleolin signal was detected that colocalized with attached microcolonies but the staining was weak and diffuse (data not shown). Because of this, we continued to use EGFP-nucleolin to study nucleolin’s behaviour during EPEC infection.

### EPEC sequesters nucleolin inside bacterial microcolonies

**Fig. 2.**
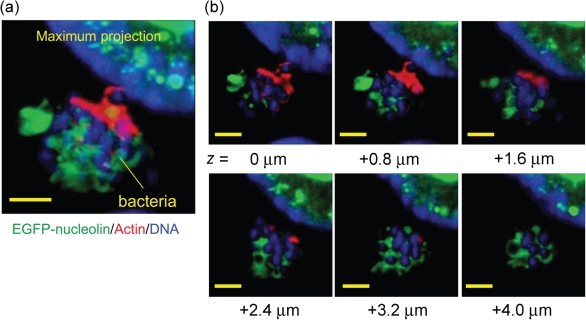
EPEC sequesters EGFP-nucleolin into its microcolonies. (a) High-resolution image showing bacterial microcolony associated with extensive EGFP-nucleolin at 120 min post-infection. (b) The bacterial microcolony in (a) was sectioned at 0.8 µm increments along the *z* axis and the images were deconvolved to reveal the EGFP-nucleolin pattern within the microcolony. Yellow scale bars, 3 µm.

To gain further insight into the nature of the cell-surface nucleolin recruitment, we performed high-magnification confocal analysis along the *z*-axis of infected cells. Remarkably, confocal sectioning through attached bacterial microcolonies revealed that the recruited EGFP-nucleolin was found, not beneath the bacteria, but throughout the microcolony, sequestered around individual bacteria (Fig. 2a). Incremental 0.8 µm *z*-axis sections (Fig. 2b) showed EGFP-nucleolin in each cross-section of the microcolony, even up to 4 µm above the level of the host cell plasma membrane, near the microcolony apex (Fig. 2b). Infection of Caco-2 cells expressing EGFP alone did not result in EGFP sequestration by the bacterial microcolonies, suggesting that the findings were specific to the recruitment of nucleolin (data not shown). A previous IF study using Hep-2 cells infected with EPEC (Sinclair & O'Brien, 2004) did not report that nucleolin was sequestered inside bacterial microcolonies, suggesting that this may not occur on non-target Hep-2 cells or that the levels of nucleolin within the microcolonies were too low to detect by IF.

### The nucleolin ligands midkine and pleiotrophin inhibit EPEC-mediated disruption of barrier function

**Fig. 3.**
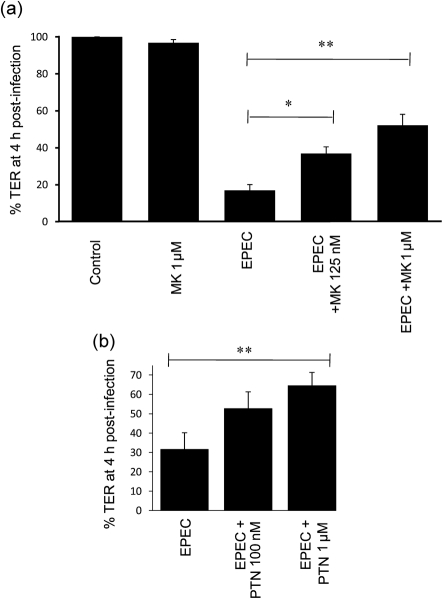
Nucleolin ligands impair the loss of TER during EPEC infection: TER of Caco-2 cells treated with or without (a) MK or (b) PTN and then infected with EPEC for 4 h. All data points represent means±sem, *n* = 3. *, *P*<0.05; **, *P*<0.01.

**Fig. 4.**
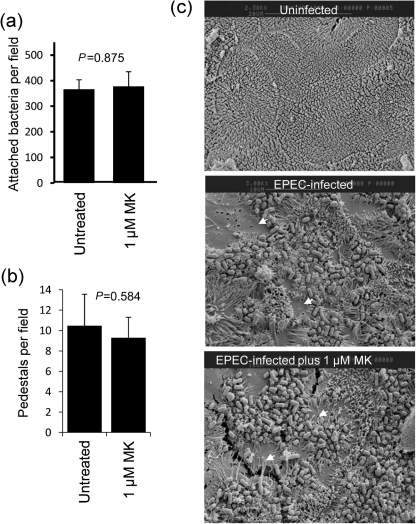
The nucleolin ligand MK does not impair bacterial attachment, pedestal formation or microvilli effacement. (a) Number of attached bacteria to Caco-2 cells after 30 min infection following incubation with or without 1 mM MK. Data points represent mean bacterial numbers per field (10 fields, *n* = 3). (b) EPEC-induced pedestals as assessed by TRITC-phalloidin were counted on Caco-2 cells with or without MK treatment. All data points in (a) and (b) represent means±sem, *n* = 3. (c) Electron microscopy of the Caco-2 cell surface infected with EPEC following treatment with or without 1 mM MK. Arrows indicate microvilli effacement.

To test the involvement of cell-surface nucleolin in EPEC infection, we used the cytokine midkine (MK) and the growth factor pleiotrophin (PTN), which bind cell-surface nucleolin (Said *et al.*, 2005; Take *et al.*, 1994) and inhibit HIV attachment to host cells (Hovanessian, 2006). We assessed whether MK or PTM exposure to Caco-2 cells would interfere with various aspects of the EPEC infection process including (i) disruption of transepithelial electrical resistance (TER; a measure of epithelial barrier function), (ii) microvilli effacement, (iii) pedestal formation and (iv) bacterial attachment. In agreement with previous studies (Dean & Kenny, 2004), EPEC caused a rapid loss of TER on Caco-2 cells (Fig. 3a) that was significantly attenuated on cells that had been incubated for 3 h with MK (Fig. 3a) or PTN (Fig. 3b) in a dose-dependent manner (*P*<0.001). Further analysis using MK revealed no significant defects in the ability of EPEC to attach to host cells (Fig. 4a, *P* = 0.875; and Fig. 4c), induce actin-based pedestals (Fig. 4b; *P* = 0.584) or cause microvilli effacement (Fig. 4c) at the same MK concentrations as used in the TER experiments. Importantly, microvilli effacement is mediated by at least three EPEC effector proteins that are delivered into the host cell (Dean *et al.*, 2006), while pedestal formation requires the effector protein Tir, suggesting that MK does not disrupt effector protein delivery. The results also show that MK had no effect on the ability of EPEC to attach to intestinal Caco-2 cells, suggesting that either MK does not efficiently block EPEC-nucleolin binding, or nucleolin may not act as an adhesin for EPEC on Caco-2 cells, unlike that shown on non-target Hep-2 cells with EHEC (Sinclair *et al.*, 2006).

## Discussion

Nucleolin is a well-studied eukaryotic protein that is important for ribosome biogenesis and represents the most abundant nucleolar protein in dividing cells (Ginisty *et al.*, 1999). It is becoming increasingly clear that nucleolin exhibits a plethora of extra-ribosomal functions, many of which occur outside the nucleolus (Ginisty *et al.*, 1999; Srivastava & Pollard, 1999; Ugrinova *et al.*, 2007). A number of pathogens have been shown to target nucleolin or cause changes in its subcellular location, an event which likely alters the cellular function of this protein. EHEC has been previously shown to recruit nucleolin to the vicinity of the bacterial infection site *in vivo* (Sinclair *et al.*, 2006). Given the similar modes of pathogenesis of the two pathogens, it was not surprising to find that EPEC recruited cell-surface EGFP-nucleolin in Caco-2 cells. However, our finding that nucleolin was transiently sequestered into the inside of extracellular bacterial microcolonies was a novel and unexpected finding. Moreover, all of the EGFP-nucleolin recruited by EPEC was inside microcolonies, with no detectable nucleolin beneath adherent bacteria that were engaged with the host plasma membrane. Further work is required to determine why nucleolin recruitment is transient and whether the bacteria actively degrade the nucleolin within the microcolony during infection.

Cell-surface nucleolin was previously shown to be an adhesin for EHEC but this may not be the case for EPEC as (a) nucleolin was found inside EPEC microcolonies and not at the interface between host cells and bacteria and (b) MK or PTN had no significant effect on bacterial attachment. Both MK and PTN were used at high concentrations that have previous been shown to saturate the nucleolin-binding sites on a range of cell types (Said *et al.*, 2002, 2005) and that also completely inhibit the nucleolin-mediated attachment of HIV to host cells. Nevertheless, it is still possible that MK may not specifically impede the binding of EPEC cells to nucleolin, and this could explain why this cytokine did not affect EPEC adherence levels.

The positive correlation between MK exposure and EPEC’s inability to disrupt epithelial barrier function suggests that nucleolin may play a role in this process, and indeed several studies have shown that cell-surface nucleolin mediates signalling pathways (Losfeld *et al.*, 2009; Reyes-Reyes & Akiyama, 2008) that may affect the integrity of the epithelial barrier. We have not ruled out the possibility that MK and PTN may have non-specific effects on the host cell, unrelated to nucleolin, that may also impede EPEC’s ability to reduce the TER without influencing type III secretion or adhesion. Thus, further work is needed to elucidate the involvement of nucleolin in this process and how the bacteria subvert its function.

In this study, and in agreement with a recent report (Dean *et al.*, 2010), we show that EPEC causes the cytoplasmic mobilization of EGFP-nucleolin from the nucleolus during late-stage infection. The recruitment of cell-surface nucleolin into bacterial microcolonies occurs earlier in infection, prior to this dramatic event. Thus, taken together, this work supports the notion that EPEC exploits two distinct pools of nucleolin during the infection process – an early cell-surface-expressed pool (this study) and a much later nucleolar/nuclear pool (Dean *et al.*, 2010) – which appear to have very different roles during the EPEC infection process. Future work may shed light on whether this versatile eukaryotic protein plays key roles in host–pathogen interactions.
